# DCE-MRI Quantitative Parameters as Predictors of Treatment Response in Patients With Locally Advanced Cervical Squamous Cell Carcinoma Underwent CCRT

**DOI:** 10.3389/fonc.2020.585738

**Published:** 2020-10-29

**Authors:** Bing Liu, Zhen Sun, Wan-Ling Ma, Jing Ren, Guang-Wen Zhang, Meng-Qi Wei, Wei-Huan Hou, Bing-Xin Hou, Li-Chun Wei, Yi Huan, Min-Wen Zheng

**Affiliations:** ^1^Department of Radiology, Xijing Hospital, Fourth Military Medical University, Xi’an, China; ^2^Department of Orthopedic, Xijing Hospital, Fourth Military Medical University, Xi’an, China; ^3^Department of Radiology, Longgang District People’s Hospital, Shenzhen, China; ^4^Department of Radiation Oncology, Xijing Hospital, Fourth Military Medical University, Xi’an, China

**Keywords:** cervical cancer, dynamic contrast-enhanced magnetic resonance imaging, concurrent chemoradiotherapy, Tofts DCE-MRI model, treatment response

## Abstract

**Purpose:**

To evaluate the predictive value of dynamic contrast-enhanced magnetic resonance imaging (DCE-MRI) quantitative parameters in treatment response to concurrent chemoradiotherapy (CCRT) for locally advanced cervical squamous cell carcinoma (LACSC).

**Methods and materials:**

LACSC patients underwent CCRT had DCE-MRI before (e0) and after 3 days of treatment (e3). Extended Tofts Linear model with a user arterial input function was adopted to generate quantitative measurements. Endothelial transfer constant (K^trans^), reflux rate (K_ep_), fractional extravascular extracellular space volume (V_e_), and fractional plasma volume (V_p_) were calculated, and percentage changes ΔK^trans^, ΔK_ep_, ΔV_e_, and ΔV_p_ were computed. The correlations of these measurements with the tumor regression rate were analyzed. The predictive value of these parameters on treatment outcome was generated by the receiver operating characteristic (ROC) curve. Univariate and multivariate logistic regression analyses were conducted to find the independent variables.

**Results:**

K^trans^-e0, K_ep_ -e0, ΔK^trans^, and ΔV_e_ were positively correlated with the tumor regression rate. Mean values of K^trans^-e0, K^trans^-e3, ΔK^trans^, and ΔV_e_ were higher in the non-residual tumor group than residual tumor group and were independent prognostic factors for predicting residual tumor occurrence. K^trans^-e3 showed the highest area under the curve (AUC) for treatment response prediction.

**Conclusions:**

Quantitative parameters at e0 and e3 from DCE-MRI could be used as potential indicators for predicting treatment response of LACSC.

## Introduction

Cervical cancer is the fourth leading cause of cancer death in women worldwide (1). Concurrent chemoradiotherapy (CCRT) is the primary choice for locally advanced cervical cancer patients, with significant benefits even for the advanced-stage disease (2). Although the alternative or novel treatment modalities could potentially improve treatment outcomes further, there is concern regarding treatment toxicity and complications in survivors (3). Moreover, even with the same clinical stage and pathological subtype, the prognosis differs among patients, which indicates tumor heterogeneity and distinct radio-sensitivity. Techniques providing composite prognostic information than current clinical prognostic factors like stage, grade, histology, and patients comorbidities would allow individualization of treatment (4). Techniques that reflect biological changes during the complex process of chemoradiotherapy are of great importance.

Tumor blood supply is normally through direct perfusion or/and vessel leakage. These vascular signatures impact radio-sensitivity by regulating the generation of oxygen free radical, which is involved in the repair of radiation-mediated DNA damage (4). Tumor vascular characteristics affect the degree of exposure to chemotherapy drugs, as well as drug activity levels *via* measuring the intra-tumor pH and the ratio of the quiescent cells in the tumor (5). As a non-invasive examining technique, MRI is widely applied worldwide and is already a standard staging protocol for cervical cancer. Dynamic contrast-enhanced magnetic resonance imaging (DCE-MRI) is regarded as a potential predictive option due to its capacity in the reflection of the perfusion by enhancement pattern, permeability, and the intratumoral angiogenic activity in the tumors (6, 7). DCE-MRI is thus applied to provide physiologic information of the tumor as well as anatomic details that are valuable for radiotherapy treatment response. These advantages make DCE-MRI an ideal tool in tumor perfusion studies that require repeated imaging.

DCE-MRI is widely applied as a non-invasive technique that plays an important role in predicting treatment response in various diseases (6, 8). Several studies have shown a correlation between DCE-MRI semiquantitative measurements and tumor response in cervical cancer patients (9). To date, there is a paucity of information in the literature about the predictive value of DCE-MRI quantitative parameters in treatment response for cervical cancer patients treated with CCRT.

The current standard treatment protocol for locally advanced cervical cancer patients is CCRT, regardless of the histological subtype of the disease. Several studies reported that patients with cervical adenocarcinoma had a poor response rate from treatment and overall survival than patients with squamous cell carcinoma (10, 11). To exclude the influence of histological subtype, we only enrolled squamous cell carcinoma patients.

This prospective study aimed to investigate the prognostic value of pre- and mid-treatment DCE-MRI quantitative parameters in the treatment prediction of patients with LACSC underwent CCRT.

## Methods and Materials

### Patients

This single-center prospective study was approved by the Ethics Committee of Xijing Hospital and informed consent was obtained from all patients. From October 2018 to April 2019, 51 consecutive cervical cancer patients administered CCRT in the department of radiation oncology were prospectively recruited. The inclusion criteria were (1) histologic diagnosis of cervical squamous cell carcinoma, (2) planned to receive CCRT in our hospital, (3) the largest diameter of the cervical tumor was 1.0 cm or larger, and (4) no contraindications to DCE-MRI. All patients conducted DCE-MRI before and 3 days after CCRT. Three patients who changed treatment regimes were excluded. Thus, the final cohort analyzes 48 LASCS patients. Clinical characteristics are presented in **Table 1**.

**Table 1:** Baseline clinical characteristics of patients (n = 48).

Characteristics	Overall (n = 48)
Age (years), median (range)	55 (29–67)
FIGO stage, n (%)	
IB	2 (4.17%)
II	34 (70.83%)
III	8 (16.67%)
IVA	4 (8.33%)
Lymph node involvement, n (%)	
Negative	29 (60.42%)
Positive	19 (39.58%)
Overall treatment duration (days), Median (range)	59 (45–71)
The interval between pretreatment DCE-MRI and initial therapy (days), Median (range)	6 (2–9)

### Concurrent Chemoradiotherapy

All patients were treated with a combination of external beam radiotherapy (EBRT) and intracavitary brachytherapy (ICBT). EBRT was delivered to the whole pelvis with 15-MV photon beams at a daily dose of 2 Gy, 5 times per week, for a total dose of 50 Gy. EBRT was accompanied by concurrent chemotherapy: six cycles of weekly cisplatin (30 mg/mm^2^) in 30 patients and three cycles of 5-fluorouracil (1,000 mg/mm^2^) plus cisplatin (60 mg/mm^2^) at 3 weeks intervals in 18 patients. ICBT was delivered twice a week in 4 fractions with a fractional dose of 7 Gy at point A. the median overall treatment time was 59 days (range 45–71 days). The selection of the chemotherapeutic regime was individualized according to local tumor extent, pelvic lymph node involvement, and general patient condition (12).

### Imaging Protocol

DCE-MRI was carried out at two time points: before the start of treatment (e0) and after 3 days of CCRT (e3). All MRI was performed at 3.0 T MRI system (Discovery MR 750, GE Medical Systems, Chicago, IL, USA) with an eight-channel phased-array Torso coil. The bladder was half-filled to improve lesion visibility. The scanning range covered the whole pelvis. Scanning parameters were as follows: axial fast spin-echo (FSE) T1-weighted images (T1WI) (repetition time[TR]/echo time [TE]: 400 ms/7.3 ms, NEX: 2, slice thickness/gap: 5 mm/1 mm, field of view [FOV]: 380 × 380 mm, acquisition matrix: 384 × 256 mm); axial and sagittal fat suppression (FS) fast spin-echo (FSE) T2-weighted images (T2WI) (TR/TE: 4000 ms/130.2 ms, NEX: 2, slice thickness/gap: 5 mm/1 mm, FOV 380 × 224 mm, acquisition matrix: 240 × 240 mm).

DCE-MRI was performed using liver acquisition with volume acceleration-extended volume (LAVA-EV) sequence (TR/TE: 6.1 ms/1.1 ms, NEX: 1, slice thickness/gap: 4 mm/−2.0 mm, FOV: 260 × 260 mm; acquisition matrix: 256 × 128 mm) (TR/TE 3.6/1.8 ms, flip angle 3°, 6°,9°,12°, slice thickness 4 mm, no interslice gap, acquisition time 5 min 31 s). Before the injection of contrast material, unenhanced images were obtained by using axial 3D spoiled gradient recalled echo sequence series with flip angles of 3°, 6°, 9°, and 12°. Before and immediately after intravenous injection of 0.1 mmol/kg Gd-DTPA (Omniscan; GE Healthcare, Shanghai, China) at a rate of 3.0 ml/s, Dynamic images were obtained from the uterine fundus to the vulva and the total acquisition time was 320 s (8 s for each phase, 40 phases). Then, the delayed contrast-enhanced MR images for axial, coronal, and sagittal planes were obtained sequentially after dynamic contrast-enhanced MR images.

### Image Analysis

Visible tumors were outlined by two radiologists on the sagittal T2WI and T1 dynamic images. Tumor volume was calculated by multiplying the area of tumor outlined on each T2WI by slice thickness. The final tumor regression rate (%) was calculated according to the following equation: 100 × (pretreatment volume − volume at 1 month after the finish of CCRT)/pretreatment volume. Previous studies have shown that the extent of the tumor regression rate correlates with survival (4).

Pharmacokinetic analysis was conducted using Omni-Kinetics (GE Healthcare, Life Science, Shanghai, China). User Arterial input function (AIF) was conducted by placing the ROI on the external iliac artery on the axial plane when reaching peak arterial enhancement (13, 14). The largest slice of the visible tumor on the axial plane was selected for arbitrarily ROI placement, which was carefully outlined around the tumor but avoiding cystic lesions with reference to T2WI, T1WI, and enhanced images. We used the Extended Tofts Linear model to generate endothelial transfer constant(K^trans^), the reflux rate (K_ep_), the fractional extravascular extracellular space (EES) volume (V_e_), and the fractional plasma volume (V_p_) (15). Based on the e0 and e3 DCE-MRI, the relative change in quantitative parameters were calculated, which were presented as Δ. Δparameter (%) was calculated based on the following equation: 100 × (parameter-e3 − parameter-e0)/parameter-e0.

### Tumor Response Assessment

Response Evaluation Criteria in Solid Tumors (RECIST 1.1V) was used for treatment response assessment. Tumor response was assessed one month after the whole CCRT, which was conducted by treating doctors from the Department of radiation oncology. Patients with complete response were classified as non-residual tumor group, while patients with partial response, stable disease, and progressive disease were classified as residual tumor group.

### Statistical Analysis

Quantitative parameters were presented as mean ± standard deviation (SD). The zero values of K^trans^ were ruled out to exclude non-perfused/necrotic regions, where the pharmacokinetic model is not applicable.

Statistical analyses were performed using SPSS (Version 17.0, SPSS Inc., Chicago, IL, USA) and GraphPad Prism 8 (GraphPad Prism Software Inc., San Diego, California, USA). Spearmen’s correlation coefficient (*r*) was conducted to assess the correlation between quantitative parameters and tumor regression rate. Mann-Whitney U test was conducted to compare parameters between residual and non-residual tumor group. The predictive value of parameters was calculated by the receiver operating characteristic curve (ROC). Univariate and multivariate logistic regression analysis was conducted to find the independent variables. A two-tailed P value of less than 0.05 was considered statistically significant.

## Results

### Patients

A total of 48 patients were eventually enrolled in this prospective study. Clinical characteristics for these patients are presented in **Table 1**. The median interval between pretreatment DCE-MRI and initial therapy was 6 days (range 2–9 days). The mean area from the ROIs was 11.2 cm^2^ (ranged 2.3–35.8 cm^2^) in the pretreatment MRI scans and 4.3 cm^2^ (range 0–8.4 cm^2^) in the I month after CCRT scans. The mean tumor volume before treatment was 45.64 cm^3^(range 11.3–192.7 cm^3^) and 19.84 cm^3^(range 0–92.84 cm^3^) at I month after CCRT. Mean tumor regression rate was 68.77% (ranged 41.77%–100%). One month after CCRT, 33 patients were categorized as non-residual tumor group and 15 patients were categorized as residual tumor group.

### Quantitative Parameters in Non- and Residual Tumor Group Patients

**Figure 1:**
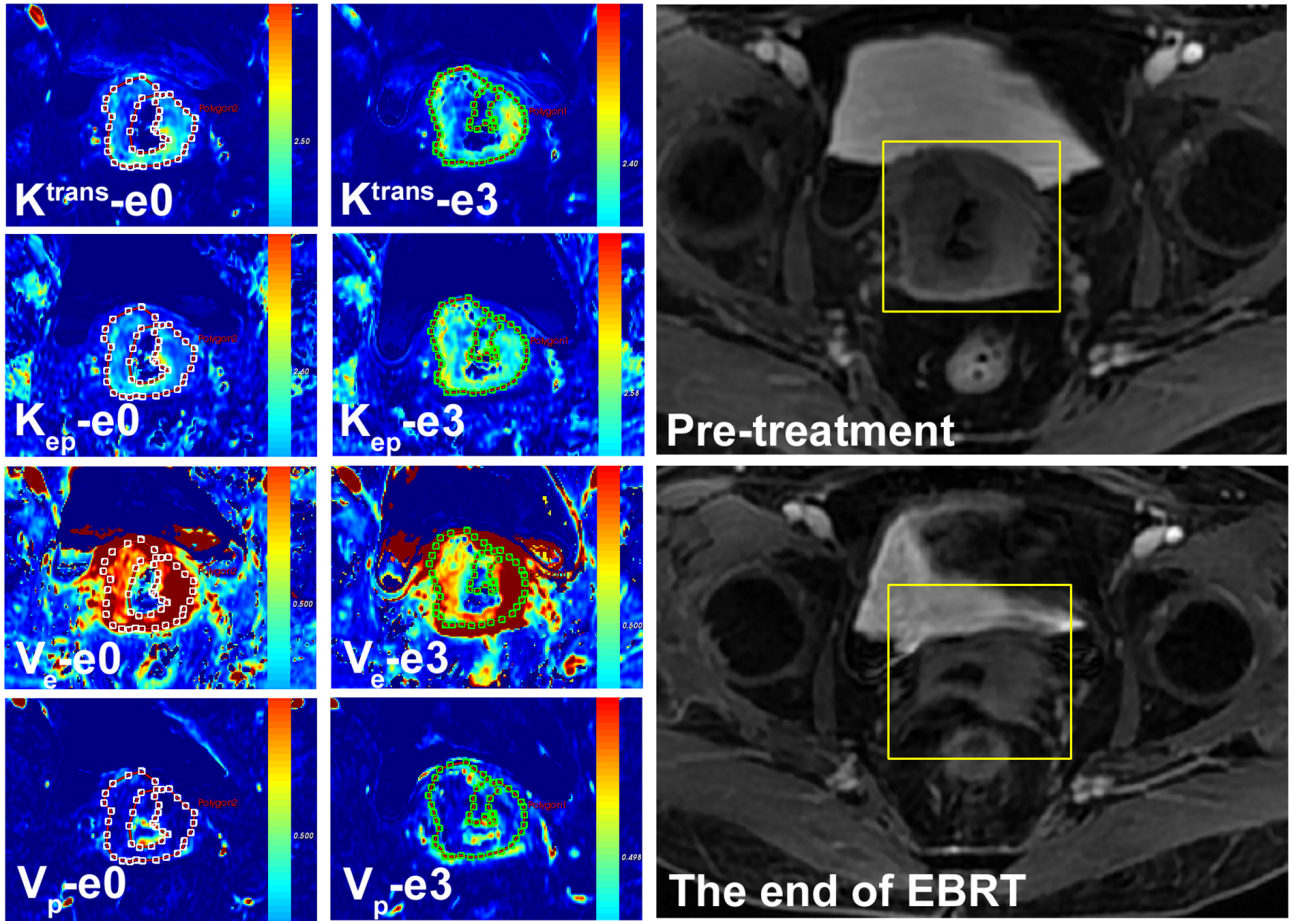
A 57-year-old female with LACSC from a non-residual tumor group. The mean value of K^trans^, K_ep_, V_e,_ and V_p_ at e0 were 1.32 min^−1^, 1.72 min^−1^, 0.66, and 0.13, respectively. The mean value of K^trans^, K_ep_, V_e_, and V_p_ at e3 were 1.53 min^−1^, 1.53 min^−1^, 0.70, and 0.15, respectively.

**Figure 2:**
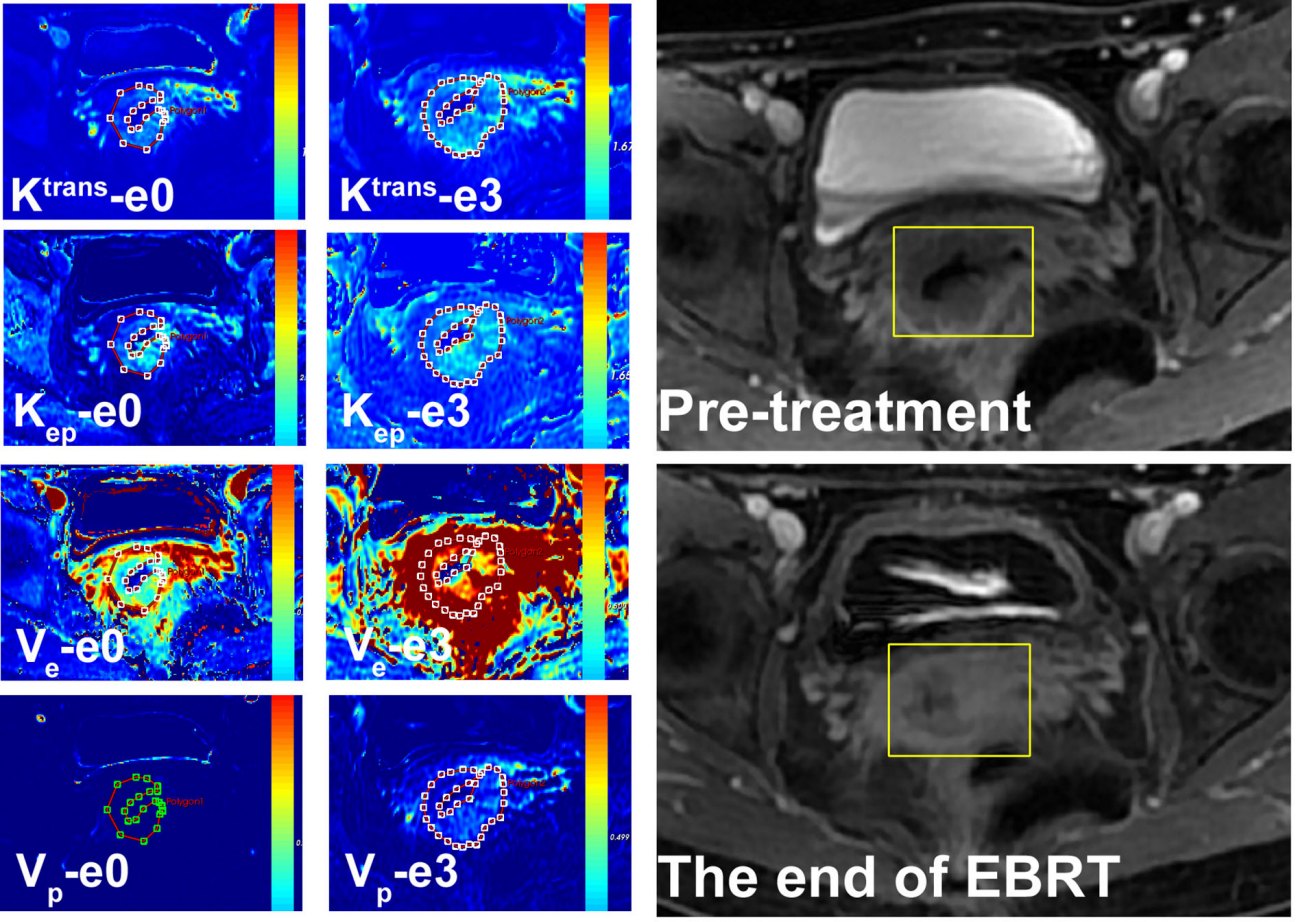
A 54-year-old female with LACSC from residual tumor group. The mean value of K^trans^, K_ep_, V_e_, and V_p_ at e0 were 1.11 min^−1^, 1.68 min^−1^, 0.63, and 0.14, respectively. The mean value of K^trans^, K_ep_, V_e_, and V_p_ at e3 were 1.32 min^−1^, 1.56 min^−1^, 0.65, and 0.14, respectively.

The correlation between quantitative parameters and treatment outcome of LACSC to CCRT are presented in **Table 2**. The non-residual tumor group had higher pre- and mid-treatment K^trans^ and V_e_ changed more significantly compared with the residual tumor group. The delayed DCE-MRI and color maps are shown in **Figures 1** and **2**.

**Table 2:** Quantitative parameters and treatment response.

Quantitative parameters	Tumor regression rate (n = 48)		Non-residual tumor group (n = 33)	Residual tumor group (n = 15)	P value
	*r* value	P value			
K^trans^-e0(min^−1^)	0.576	<0.001^*^	1.36 ± 0.33	1.12 ± 0.35	0.04^*^
K_ep_-e0(min^−1^)	0.528	<0.001^*^	1.74 ± 0.39	1.67 ± 0.33	0.25
V_e_ -e0	0.434	0.159	0.64 ± 0.22	0.64 ± 0.20	0.94
V_p_-e0	0.386	0.216	0.14 ± 0.08	0.13 ± 0.09	0.99
K^trans^-e3(min^−1^)	0.617	0.025^*^	1.58 ± 0.47	1.29 ± 0.46	0.02^*^
K_ep_-e3(min^−1^)	0.584	0.194	1.59 ± 0.43	1.53 ± 0.38	0.06
V_e_ -e3	0.403	0.195	0.71 ± 0.17	0.63 ± 0.25	0.09
V_p_-e3	0.261	0.467	0.13 ± 0.11	0.15 ± 0.10	0.63
ΔK^trans^ (%)	0.507	<0.001^*^	41.54 ± 34.31	31.44 ± 33.15	0.04^*^
ΔK_ep_ (%)	0.410	0.186	−4.75 ± 30.36	−5.40 ± 28.67	0.75
ΔV_e_ (%)	0.542	<0.001^*^	25.80 ± 52.02	6.96 ± 46.94	0.01^*^
ΔV_p_ (%)	0.345	0.328	36.1 ± 64.32	57.75 ± 53.45	0.08

*p < 0.05.

**Figure 3:**
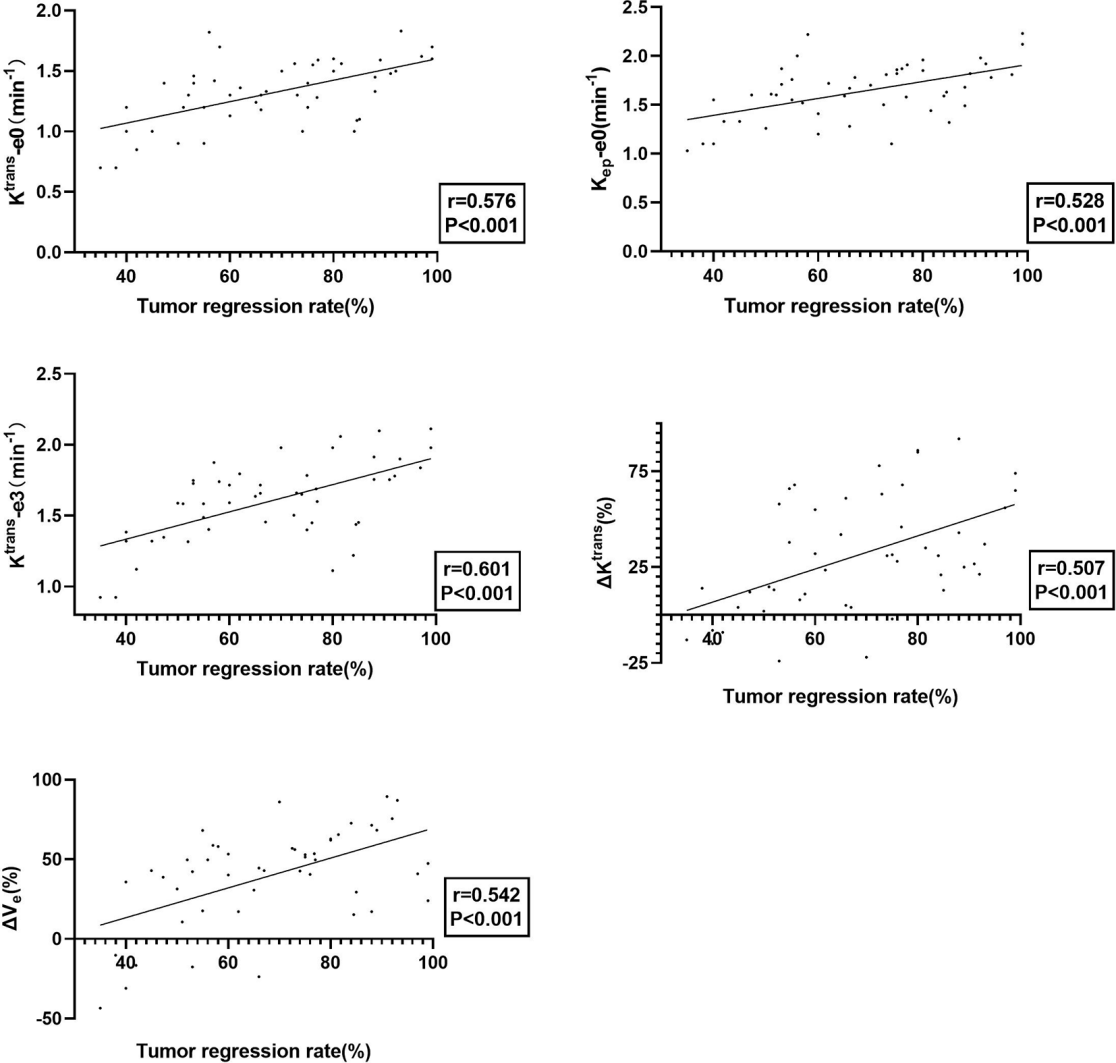
The plot of K^trans^-e0, K_ep_ -e0, K^trans^-e3, ΔK^trans^, and ΔV_e_ with tumor regression rate, showing that tumors with better treatment response exhibited better permeability and perfusion.

Tumor regression rate was positively correlated with K^trans^-e0 (*r*=0.576, P<0.001), K_ep_-e0 (*r* = 0.528, P < 0.001), K^trans^-e3 (*r* = 0.617, P = 0.025), ΔK^trans^ (*r* = 0.507, P < 0.001) and ΔV_e_ (*r* = 0.542, P < 0.001). Details are presented in **Figure 3**.

### The Receiver Operating Characteristic Curve, Univariate, and Multivariate Logistic Regression Analysis of Quantitative Parameters

**Figure 4:**
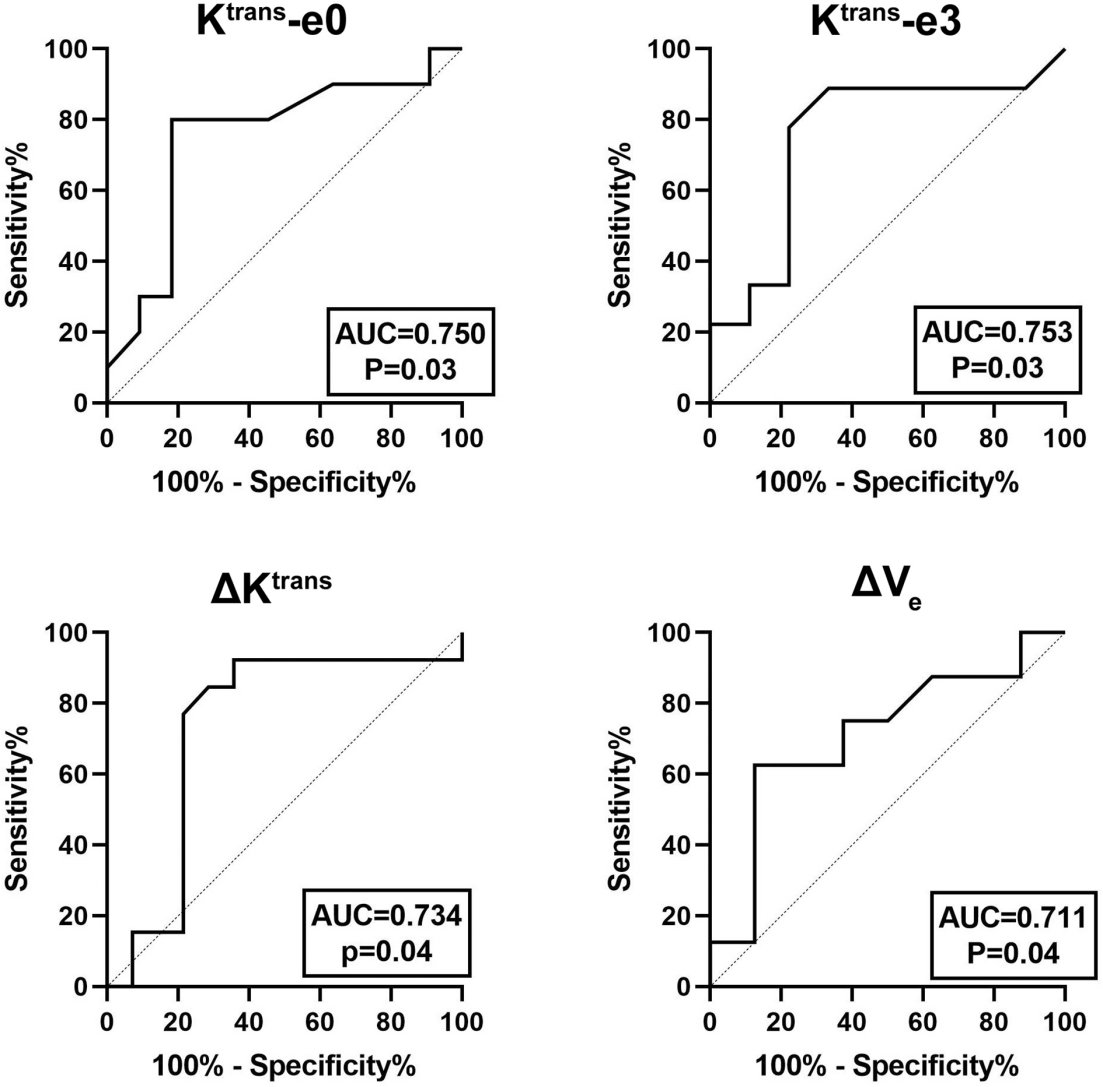
ROC curves for predicting residual tumor occurrence based on K^trans^-e0, K^trans^-e3, ΔK^trans^, and ΔV_e_. The area under curve (AUC) was 0.750, 0.753, 0.734, and 0.711, respectively.

The quantitative parameters exhibited good prognostic value to predict residual tumor occurrence, which was further validated by the ROC as presented in **Table 3** and **Figure 4**. K^trans^-e3 showed the best predictive ability. When setting cut-off value of K^trans^-e3 to 1.48, the sensitivity, specificity, PPV and NPV were 81.82%, 80.00%, 81.82%, and 80.00%, with area under curve (AUC) of 0.753 (P = 0.04).

**Table 3:** Values of quantitative parameters for predicting residual tumor occurrence.

	AUC	Cut-off	Sensitivity(%)	Specificity(%)	PPV(%)	NPV(%)	P value
K^trans^-e0 (min^−1^)	0.750	1.21	87.50	72.50	92.11	50.00	0.03^*^
K^trans^-e3 (min^−1^)	0.753	1.48	81.82	80.00	81.82	80.00	0.04^*^
ΔK^trans^ (%)	0.734	45.57	72.22	70.43	79.63	71.48	0.04^*^
ΔV_e_ (%)	0.711	36.4	77.78	66.67	82.35	60.00	0.02^*^

AUC, area under the curve; PPV, positive predictive value; NPV, negative predictive value; *p < 0.05.

Univariate and multivariate logistic regression analysis revealed that K^trans^-e0, K^trans^-e3, and ΔV_e_ were independent prognostic factors for residual tumor occurrence. The lower K^trans^-e0, K^trans^-e3, ΔK^trans^, and ΔV_e_ had higher risk ratios for residual tumor occurrence. Details are presented in **Table 4**.

**Table 4:** Univariate and multivariate logistic regression analysis for predicting residual tumor occurrence.

Parameters	OR	P value	95%CI
Univariate analysis			
K^trans^-e0 (min^−1^)	15.6	0.03^*^	1.53–104.13
K^trans^-e3 (min^−1^)	25.4	<0.01^*^	5.74–78.31
ΔK^trans^ (%)	7.9	0.02^*^	1.36–34.52
ΔV_e_ (%)	6.5	0.03^*^	1.07–29.84
Multivariate analysis			
K^trans^-e0 (min^−1^)	18.00	<0.01^*^	1.74–114.40
K^trans^-e3 (min^−1^)	21.00	<0.01^*^	4.89–68.42
ΔK^trans^ (%)	5.1	0.01^*^	2.34–11.72
ΔV_e_ (%)	7.00	0.04^*^	1.11–32.30

OR, odds ratio; CI, confidence interval; *p < 0.05.

## Discussion

CCRT is the primary option for the management of local advanced cervical cancer. Due to tumor heterogeneity, different curative responses were found with the same treatment regimen. Early knowledge of treatment response is clinically significant and enables modification of treatment regimen in early the stage of applied treatment, which prevents unnecessary toxicity or prolonged ineffective consequence in treatment resisted patients.

In the present study, DCE-MRI was used to investigate the possibility to predict short-term response to CCRT and to improve diagnostic potency in cervical squamous cell carcinoma patients. Studies have shown that cervical squamous cell carcinoma had a better response rate from CCRT, took shorter time to achieve complete response, had better overall survival, and disease-free survival than adenocarcinoma (10, 16). Thus, we only enrolled patients with cervical squamous cell carcinoma, to exclude the influence by histology type.

Tumor regression rate was chosen as a short-term endpoint due to its close relationship with local control and outcome of cancer management (17, 18). Here, we found that quantitative DCE-MRI parameters K^trans^-e0, K_ep_-e0, K^trans^-e3, ΔK^trans^, and ΔV_e_ positively correlated with tumor regression rate in LACSC. Accumulating evidence has shown the correlation between DCE-MRI parameters and tumor regression. Several studies concluded that pretreatment parameters K^trans^ and K_ep_ were positively correlated with tumor regression rate (4, 19, 20), which is inconsistent with our results. However, Park et al. reported that pretreatment K^trans^ positively correlated with tumor volume at 4 weeks of initiating CCRT (21). To our knowledge, K^trans^ and K_ep_ reflect the permeability of tumor tissue, this property has a positive influence on oxygenation within tumor tissue, which results in higher radiation sensitivity. What’s more, hypoxia is a known cause of clinical radioresistance for cervical cancer (22). Therefore, tumors with higher permeability tend to respond to better treatment outcomes and thus lead to higher tumor regression rate.

Additionally, our results revealed that mid-treatment K^trans^-e3, and the increase of K^trans^ positively correlated with tumor regression rate as well. The non-residual tumor group showed higher K^trans^ at e3, together with ΔK^trans^. Mid treatment K^trans^ value represents early treatment response to CCRT, which could be interpreted as K^trans^ reflect the effectiveness of oxygen and chemotherapy drugs between plasma and interstitial space of the tumor. Higher mid-treatment K^trans^ value can be explained as increasing levels of permeability due to the disintegration of tumor cell and capillary membranes, which are a consequence of chemoradiotherapy.

V_e_ reflects the extravascular extracellular space, and lower V_e_ value could be interpreted as higher cellularity (23). The increase of V_e_ may reflect the enlargement of fractional EES, which indicates the decrease in cell density. Several studies concerning V_e_ and its correlation with treatment response showed different results. Ellingsen et al. reported that pretreatment V_e_ showed no association with hypoxia in cervical cancer xenografts (24). Kim et al. showed that the early increase of V_e_ was associated with tumor regression in cervical cancer patients underwent CCRT (25). Also, Cheng et al. reported similar results in lung carcinoma that an early increase in V_e_ is correlated with tumor control (26). The above studies supported our results. However, Park et al. reported that pretreatment V_e_ negatively correlated with tumor volume at 1 month after the end of treatment (21). Their results suggested that higher pretreatment K^trans^ and lower pretreatment V_e_ tended to result in a larger tumor volume at 4 weeks of CCRT. They argued this was caused by secondary inflammation related to ongoing treatment. We hold the opinion that V_e_ represents a direct estimation of EES to which a contrast agent or drug can be delivered. The increase of V_e_ was the result of a decrease of tumor cell density and enlargement of the distribution space, which enables more contrast agent and chemotherapy drugs to be delivered, leading to a higher tumor regression rate.

DCE-MRI parameters have been extensively used in predicting tumor response to radiotherapy and chemotherapy (27), to non-invasively investigate tumor microvascular structure and heterogeneity, thus providing additional information to potentially improve sensitivity to the treatment regimen. We incorporated two time-points to investigate if DCE-MRI quantitative parameters can predict treatment response, and revealed that K^trans^-e0, K^trans^-e3, ΔK^trans^, and ΔV_e_ could be biomarkers to predict treatment response for LACSC. Several studies are partially consistent with our results. Tao et al. reported that pretreatment K^trans^ and K_ep_ were significantly higher and V_e_ were lower in responders than the non-responder group in non-small cell lung cancer (19). Kim et al. found in breast cancer pretreatment DCE-MRI parameters showed no difference, while after two cycles of NACT, the change of K^trans^ and K_ep_ were significantly higher in good responder group (28). Wong et al. reported in advanced head and neck cancer that larger fractional increase in K^trans^ and V_e_ in responders versus non-responders at week 2 of treatment (29). Several studies are showing conflicting results. Andersen et al. reported that pretreatment K^trans^ and V_e_ were positively correlated with progression-free survival for cervical cancer patients (30). Zheng et al. reported that K^trans^ was higher and V_e_ was lower in non-residual group cervical cancer patients (31). Semple et al. proved that pretreatment K^trans^ correlated with the response in cervical cancer patients (32). We found that K^trans^-e0, K^trans^-e3, ΔK^trans^, and ΔV_e_ are significantly higher in the non-residual tumor group than the residual tumor group in LACSC. The parameters K^trans^-e0, K^trans^-e3, and ΔK^trans^ were significantly higher in non-residual tumor group patients, which supported the hypothesis that better permeability represented better material exchange, thus better oxygenation and higher radiation sensitivity (33). Low oxygen level within tumor tissue, i.e., hypoxia causes therapeutic resistance *via* reducing the generation of oxygen free radical, which interferes with the repair of DNA damage induced by radiotherapy, thus transferring tumor cells into subtypes with more resistance to treatment regimens (34, 35). The non-residual tumor group showed higher permeability before and early during treatment, resulting in higher radio-sensitivity and access to chemotherapeutic drugs. Tumors with a higher level of perfusion and permeability are likely to be better oxygenated and therefore more sensitive to radiation. Moreover, higher perfusion also results in a higher concentration of chemotherapy drugs within the tumor. Evidence showed that low V_e_ was negatively correlated with progression-free survival, indicating that patients with high cell density had a more aggressive disease, which also supported our results.

Finally, we showed that day 3 of CCRT could be a time point to detect treatment response using DCE-MRI quantitative parameters. To our knowledge, this is the first study investigating the complementary value of DCE-MRI quantitative parameters on the 3rd day of CCRT for response prediction in patients with LACSC. Previous studies applied the time point of week 1, week 2, or week 4 (34, 35). Early detection of treatment response is important since it can avoid unnecessary toxicity and treatment-related complications. Early prediction of response during treatment may enable early modification of treatment (i.e., radiation dosage intensification or discontinuation) (28). We noticed that AUC of mid-treatment K^trans^-e3 was higher than pretreatment K^trans^-e0, probably indicated that the closer relation between mid-treatment parameter and treatment outcome.

There are several limitations in this single-center retrospective study. Firstly, the follow-up period was short, and overall survival or progression-free survival were not analyzed. Thus, we did not evaluate the correlation between pre- or mid-therapy DCE-MRI parameters and those clinical endpoints. Secondly, more time points should be set to get a full view of the dynamic change of quantitative parameters during the whole treatment process. Thirdly, further investigation should be done to investigate the correlation between oxygenation and treatment response.

In conclusion, our preliminary results showed that quantitative parameters at e0 and e3 from DCE-MRI could be used as a potential indicator for predicting treatment response of LACSC. The mean value of K^trans^-e0, K^trans^-e3, ΔK^trans^, and ΔV_e_ is potentially applicable for treatment response prediction. K^trans^-e0, K_ep_ -e0, ΔK^trans^, and ΔV_e_ can be used for predicting tumor regression rate. Further studies are needed to clarify the possibility to detect heterogeneity directly by MRI techniques. Our study therefore strongly suggests that DCE-MRI may be a useful tool for individualizing therapy of LACSC.

## Data Availability Statement

All datasets presented in this study are included in the article/supplementary material.

## Ethics Statement

The studies involving human participants were reviewed and approved by Ethics Committee of Xijing Hospital. The patients/participants provided their written informed consent to participate in this study. Written informed consent was obtained from the individual(s) for the publication of any potentially identifiable images or data included in this article.

## Author Contributions

YH and M-WZ conceived and designed this study. JR, M-LM, and W-HH conducted the study. B-XH and L-CW collected important background data. BL and ZS drafted the manuscript. All authors contributed to the article and approved the submitted version.

## Funding

This work was supported by Chinese National Natural Science Foundation Grants (No. 81220108011).

## Conflict of Interest

The authors declare that the research was conducted in the absence of any commercial or financial relationships that could be construed as a potential conflict of interest.
